# Antibacterials in Aquatic Environment and Their Toxicity to Fish

**DOI:** 10.3390/ph13080189

**Published:** 2020-08-09

**Authors:** Bartosz Bojarski, Barbara Kot, Małgorzata Witeska

**Affiliations:** 1 Department of Zoology and Animal Welfare, Faculty of Animal Science, University of Agriculture in Krakow, Mickiewicza 24/28, 30-059 Krakow, Poland; 2 Institute of Biological Sciences, Faculty of Exact and Natural Sciences, Siedlce University of Natural Sciences and Humanities, Prusa 14, 08-110 Siedlce, Poland; barbara.kot@uph.edu.pl (B.K.); malgorzata.witeska@uph.edu.pl (M.W.)

**Keywords:** antibiotics, chemotherapeutics, toxic effects, oxidative stress, hematological changes, histopathology, antibiotic resistance

## Abstract

Antibacterial agents are commonly present in aquatic environment at low concentrations. Terrestrial animal farms, human medicine and aquaculture are main sources of water contamination with antibacterials. Antibiotics were proved to be directly toxic to fish causing oxidative stress, general stress response, histopathological lesions, hematological, metabolic, and reproductive disorders, as well as immunosuppressive and genotoxic effects. Environmentally realistic low concentrations of antibiotics also disturb aquatic bacterial communities causing alterations in fish symbiotic microbiota and induce emergence of antibiotic-resistant pathogenic bacteria by exerting selective pressure on spread of antibiotic-resistance genes.

## 1. Introduction

Antibacterial agents are commonly used to control bacterial diseases in both human and veterinary medicine. They include various bacteriostatic or bactericidal compounds such as antibiotics (substances produced by microorganisms or their synthetic analogs) and chemotherapeutics (artificial antibacterial agents that do not occur in nature). Due to their misuse, overdose, and poor absorption accompanied by high water solubility and resistance to degradation, antimicrobials are commonly found in environment [1,2]. Intensive livestock production is currently a main source of environmental antimicrobial load. According to Landers et al. [3], 24.6 million pounds of antimicrobials are used for nontherapeutic (prevention and growth promotion) purposes in chickens, cattle, and swine, compared with just 3.0 million pounds used in human medicine. Analysis of antimicrobial use in France from 1999 to 2005 revealed that in human medicine mainly β-lactams and macrolides are used, while in veterinary-tetracyclines, sulfonamides, and trimethoprim predominated [4]. Antimicrobials used in animals cannot be completely absorbed or metabolized in the organisms to which they have been administered, and about 30–90% of the used amount is excreted through urine and feces and released into the surrounding ecosystems [5]. According to Letsinger and Kay [6], only a small part of human pharmaceuticals have been monitored in aquatic environment and little is known about their effects on organisms. Large amounts of veterinary antimicrobials reach agricultural fields due to manure fertilization [7,8]. Thus, livestock manure is an important source of environment contamination with antimicrobial compounds [9]. Martinez-Carballo et al. [10] reported that in Austria pig manure contained up to 46 mg/kg of chlortetracycline, 29 mg/kg of oxytetracycline, and 23 mg/kg of tetracycline. Enrofloxacin was particularly observed in chicken and turkey samples. According to Zhao et al. [11], analysis of 61 pig, 54 chicken, and 28 cow manure samples collected in China revealed that pig manure contained up to 33.98 mg/kg ciprofloxacin, 33.26 mg/kg enrofloxacin, 59.06 mg/kg oxytetracycline, 21.06 mg/kg chlortetracycline, cow manure up to 29.59 mg/kg ciprofloxacin, 46.70 mg/kg enrofloxacin, 59.59 mg/kg oxytetracycline, and 27.59 mg/kg chlortetracycline, while in chicken manure 99.43 mg/kg fleroxacin, 225.45 mg/kg norfloxacin, 45.59 mg/kg ciprofloxacin, and 1420.76 mg/kg enrofloxacin was detected. According to Hu et al. [12], winter manure contained higher levels of various antibiotics (with maximum of 183.5 mg/kg oxytetracycline) than summer manure (up to 29.3 mg/kg tetracycline). Antibiotics released from manures can enter the soil, followed by ground and surface water. The presence of antibiotics in aquatic environment is worrying not only because of the possibility of the emergence of resistant bacterial strains [13], but also in the context of possible toxic effects on aquatic organisms, including fish.

The aim of the present review was to summarize available data on antibacterials present in aquatic environment, discuss the phenomenon of antibiotic resistance associated with them, but above all, analyze the toxic effects in fish that may occur due to contamination of aquatic environment with antibacterial drugs. These issues are relevant to protecting the aquatic environment from the pollution as well as in terms of the health of fish. 

## 2. The Presence of Antibacterials in Aquatic Environment

The occurrence and fate of pharmaceutically active compounds in aquatic environment has been recognized as one of the emerging issues in environmental chemistry [14]. Awad et al. [5] observed high concentrations of tetracyclines and sulfonamides in river water close to a swine manure composting facility in Korea. Considerable seasonal variation was reported: in June water contained up to 46 µg/L of tetracycline and 4.6 µg/L of sulfathiazole, while in September 255 and 10.6 µg/L, respectively. Wei et al. [15] examined animal wastewater and surface water around large-scale livestock and poultry farms in China. They monitored 53 samples collected from 27 large-scale animal farms in 11 cities and counties of Jiangsu Province. Ten veterinary antimicrobial compounds were detected in wastewaters, 8 of them were found in pond waters, and animal farm-effluents and river water samples were contaminated by 9 compounds. The most frequently detected antimicrobials were sulfamethazine (75%), oxytetracycline (64%), tetracycline (60%), sulfadiazine (55%), and sulfamethoxazole (51%), with a maximum concentrations of 211, 72.9, 10.3, 17.0, and 63.6 µg/L, respectively. Zhou et al. [16] investigated the occurrence of 4 classes of 17 commonly used human and veterinary antibiotics in the sediments of the Yellow River, Hai River, and Liao River (China). Norfloxacin, ofloxacin, ciprofloxacin, and oxytetracycline were most frequently detected in all rivers studied, with concentrations up to 5770, 1290, 653, and 652 µg/L, respectively. According to Cui et al. [17], drinking water source reservoir in the Yangtze River delta contained 12 antimicrobial compounds belonging to 4 classes (sulfonamides, fluoroquinones, tetracyclines, and macrolides). Cheng et al. [18] reported the presence of quinolones, tetracyclines, amphenicols, and sulfonamides at concentrations 0.2–421 ng/L in river water samples from North China. Li et al. [19] reviewed the occurrence of antimicrobials in water and sediments from 7 major rivers and 4 seas of China. They reported frequent presence of 12 compounds belonging to sulfonamides, tetracyclines, fluoroquinolones, and macrolides at median concentrations under 100 ng/L in water and 100 ng/g in the sediments. According to Liu et al. [20], China is the world largest producer and exporter of aquatic food products and antimicrobials are widely applied in aquaculture. A total of 20 antibacterial agents belonging to 8 groups (aminoglycosides, β-lactams, chloramphenicols, macrolides, nitrofurans, quinolones, sulfonamides, and tetracyclines) were used in 1996–2013, among them 12 compounds were not authorized. Teglia et al. [21] observed 0.97–22.1 µg/L of various fluoroquinolone antibiotics (ciprofloxacin, enrofloxacin, ofloxacin, enoxacin, and difloxacin) in farm wastewaters of various regions in Argentina. Gbylik-Sikorska et al. [22] investigated the occurrence of commonly used veterinary antibacterial agents in 159 fresh water, 443 fish, and 150 sediment samples from Polish rivers and lakes. This study showed the presence of aminoglycosides, β-lactams, diaminopyrimidines, fluoroquinolones, macrolides, lincosamides, pleuromutilins, sulfonamides, and tetracyclines. Sacher et al. [23] reported the occurrence of sulfamethoxazole (maximum concentration 410 ng/L) and anhydro-erythromycin (the degradation product of erythromycin; up to 49 ng/L) in groundwater samples collected in Germany. Managaki et al. [24] found 7 human-used antimicrobials: sulfamethoxazole, sulfapyridine, trimethoprim, erythromycin-H_2_O, azithromycin, clarithromycin, and roxithromycin at concentrations 4–448 ng/L in the urban Tamagawa River, Japan, and sulfamethoxazole, sulfamethazine, trimethoprim, and erythromycin-H_2_O (7–360 ng/L) in urban and rural areas of Mekong Delta, Vietnam. The authors also showed the presence of sulfamethazine at high concentrations (19.2 and 18.5 µg/L) in a pig farm wastewater.

According to Bielen et al. [25], antimicrobial compounds may appear in effluents of the pharmaceutical industries. They observed macrolide antibiotic azithromycin and macrolide by-products (up to 10.5 mg/L) in the treated wastewater from a Croatian antibiotic-producing company and concentrations up to 30 µg/L) in the recipient river. In effluents of another pharmaceutical factory fluoroquinolones, trimethoprim, sulfonamides, and tetracyclines to about 200 µg/L were observed and concentrations of these compounds from below the limit of quantification to a range of µg/L were measured in the recipient stream. High frequency of bacteria resistant to azithromycin (up to 83%), sulfamethazine (up to 90%) and oxytetracycline (up to 50%) were also found in the effluents. 

Aquaculture itself is also a source of environmental contamination with antimicrobials. They are commonly used in modern aquaculture to prevent or treat fish bacterial diseases that result from fish rearing at high stocking densities that induce stress, suppress immune system and facilitate pathogen transmission. The issue of use of antimicrobial drugs in aquaculture and related resistance of ichthyopathogenic bacteria was studied already over 20 years ago [26]. Development of aquaculture industry resulted in the widespread use of antimicrobials, especially in developing countries, to prevent or treat bacterial infections in fish [27]. The use of large amounts of various antibiotics resulted in the emergence of antibiotic-resistant bacteria in aquatic environments and in alterations of the aquatic bacterial communities. Andrieu et al. [28] reported that effluents of Pangasius catfish farm in Vietnam contained 0.68 µg/L of enrofloxacin and 0.25 µg/L of ciprofloxacin. Antibiotics accumulated in sediments down-stream the effluent discharge at concentrations up to 2590 µg/kg d.w. (dry weight) and 592 µg/L d.w., respectively. Miranda et al. [29] reported that Chilean salmon farms in 2005–2016 used 0.31–0.64 kg of antibiotics per harvested ton of fish. In 2005 mainly flumequine (~40%), oxytetracycline (~25%), oxolonic acid (~20%), and florfenicol (~15%) were used, while in 2016 mainly florfenicol (>80%) and oxytetracycline (<20%). According to Holmstrom et al. [30], antibacterial agents (tetracyclines, quinolones, sulfonamides, and other) were commonly used in Thai shrimp farming to treat diseases and for prophylaxis. The issues of the presence of antibacterial drug residues in the edible tissues of treated fish, environmental hazards, and development of resistant bacteria are considered a serious concern [31]. According to Liu et al. [32], tissue levels of sulfonamides, trimethoprim, fluoroquinolones, and macrolides in 7 wild fish species from Laizhou Bay (North China) in 2016 ranged from 22 to 500 ng/g dw. Conti et al. [33] revealed illegal antimicrobials: crystal violet, chloramphenicol, gentamicin, fluoroquinoloneenrofloxacin, malachite green, and the metabolites of furaltadone and furazolidone in the samples of feed and fish from a fish farm in eastern Sicily, Italy. According to the authors, illegal antimicrobials are widely used in aquaculture all over the world.

The data summarized in Table 1 (most of them from China [15,16,17,18,34,35,36,37]) show that 11 groups of antibacterials were found in aquatic environments. Among them, sulfonamides were the most commonly reported (33% of data), followed by fluoroquinolones (23%), tetracyclines (18%), macrolides (12%), diaminopyrimidines (7%), and phenicol antibiotics (2.6%). Single reports are available on the presence of aminoglycosides, β-lactams, lincosamides, and pleuromutilins.

Sulfonamides are broad spectrum chemotherapeutics, effective against Gram-positive and some Gram-negative bacteria and commonly applied in both human and veterinary medicine [38]. According to Hruska and Franek [39], sulfonamides are often used in swine and cattle farms to treat bacterial diseases, and thus such farms may be a source of these compounds in aquatic environment. Fluoroquinolones are relatively new synthetic antibiotics showing a broad spectrum and potent bactericidal activity against various clinically important pathogens [40]. According to Sarkozy [41], fluoroquinolones are used in humans to treat a variety of severe infections located in tissues inaccessible or caused by bacteria resistant to other antimicrobials. Many fluoroquinolone compounds are also approved for use in animals and applied in swine, cattle, and poultry farming being main source of water contamination. Quinolone antibiotics are also used in treatment of bacterial diseases in fish [42]. Tetracyclines are broad-spectrum antibiotics active against various Gram-positive and Gram-negative bacteria and protozoans. Versatility and high antimicrobial activity accompanied by the absence of major adverse side effects has led to their extensive use for treatment of human and animal infections. Tetracyclines are widely applied for the treatment of infections in poultry, cattle, sheep, swine, and domestic pets [43]. In the European Union tetracycline preparations are registered for use in cattle, pig, sheep, goat, horse, dog, cat, poultry, rabbit, and fish [44]. According to Jelic and Antolovic [45], macrolides are natural (produced by *Streptomyces* sp.) and newly-synthesized cyclic peptide compounds, the most commonly used class of antibiotics. Diaminopyrimidines (e.g., trimethoprim) are synthetic, broad-spectrum antimicrobial agents, mainly used in treatment of human urinary tract infections [46]. According to Kolar et al. [47], trimethoprim is also used in veterinary medicine for pigs and poultry. Phenicol compounds (chloramphenicol and its derivatives) are synthetic broad-spectrum bacteriostatic and bactericidal agents effective against many Gram-negative and Gram-positive organisms, used in human and veterinary medicine [48,49]. Aminoglycosides are natural or semisynthetic antibiotics derived from actinomycetes, active against various Gram-positive and Gram-negative organisms. They were one of the first groups of antibiotics applied for the clinical use [50]. Aminoglycosides used in veterinary medicine are effective in treating cattle bacterial enteritis and mastitis and used in prophylaxis or as growth promoters. However, aminoglycoside use as growth promoters is illegal in the European Union [51]. β-lactam antibiotics include numerous compounds being the most widely used class of antimicrobials due to their broad antibacterial spectrum and low toxicity [52]. They are used in both human and veterinary medicine [4]. According to Rezanka et al. [53], lincosamides are bacteriostatic or, at higher concentrations, bactericidal antibiotics active against Gram-positive bacteria and protozoans. Lincosamides and macrolides are used for treatment of swine gastrointestinal and respiratory infections and cattle respiratory disease [54]. Pleuromutilins include diterpene antibiotics derived from basidiomycetes and semisynthetic compounds, active against anaerobic, and some Gram-positive and Gram-negative bacteria. Most of them are used exclusively in veterinary medicine for treatment of bacterial gastrointestinal and respiratory infections in pigs and poultry, while they are not approved for ruminants or horses [55]. From 2007, pleuromutilin retapamulin is used also in humans [56].

Sulfonamides (at least 12 compounds) were observed in natural waters at the concentrations from undetectable or several ng/L to over 10 µg/L (Table 1). The highest concentrations (over 200 µg/L) were measured in effluents from terrestrial animal production or pharmaceutical industry. Little data are available on concentrations of sulfonamides in sediments (0.28–5 µg/kg).

Concentrations of fluoroquinolones (at least 7 compounds) in natural waters reported by various authors ranged from nondetectable to 5.7 µg/L, while in wastewaters up to 98 µg/L. Fluoroquinolones were also found in sediments at concentrations from below the detection limit to about 2.6 mg/kg. 

Tetracyclines (at least 4 compounds) were commonly observed in natural waters at the concentrations ranging from several ng/L to even over 254 µg/L, while in wastewaters up to 72.9 µg/L. Tetracyclines were also found in the sediments at the levels up to 625 µg/kg.

The observed levels of macrolide antibiotics (at least 6 compounds) in natural waters ranged from a few ng/L up to 5 µg/L. Wastewaters from pharmaceutical industries contained even over 5.6 mg/L of macrolides, while the sediments up to 67.7 µg/kg.

Trimethoprim was also often detected in aquatic environments at the concentrations up to 380 ng/L in natural waters and to 5.6 µg/L in pharmaceutical effluents, while the maximum level noted in the sediment was 9.84 µg/kg.

Single reports concerning the levels of other antimicrobials in water include 3 phenicol compounds present in river water (to 5.8–1.8 µg/L), aminoglycosides (up to 10 µg/L in natural freshwaters), diaminopyrimidines (<0.05 µg/L in water and <5 µg/kg in sediment), lincosamides (<0.02 µg/L in water), pleuromutilins (<0.02 µg/L), and β-lactams (to 10 µg/L in water and to 8 µg/kg in sediment).

These data showed that fluoroquinolones reached the highest levels in the sediments, while tetracyclines in the water. 

**Table 1:** The occurrence of antibacterials in the aquatic environment.

Group	Antibacterial Agents	Maximum Concentration	Sample Type/Source	Site/Region	Country	Reference
A	aminoglycosides	10 µg/L ND	fresh water sediments	14 sampling points in Polish rivers and lakes	Poland	Gbylik-Sikorska et al. [22]
BL	β-lactams	10 µg/L 8 µg/kg	fresh water sediments	14 sampling points in Polish rivers and lakes	Poland	Gbylik-Sikorska et al. [22]
D	diaminopyrimidines	<0.05 µg/L <5 µg/kg	fresh water sediments	14 sampling points in Polish rivers and lakes	Poland	Gbylik-Sikorska et al. [22]
D	trimethoprim	det 5.63 µg/kg 9.84 µg/kg	sediments sediments sediments	Yellow River Hai River Liao River	China	Zhou et al. [16]
D	trimethoprim	≈60 ng/L ≈50 ng/L	Tamagawa River (water samples) Mekong Delta (water samples)	Tokyo metropolitan area suburbs and rural areas	Japan Vietnam	Managaki et al. [24]
D	trimethoprim	4.41 ng/L	drinking water	Yangtze River delta	China	Cui et al. [17]
D	trimethoprim	5.4 ng/L	river water	Beijing-Tianjin-Hebei region	China	Cheng et al. [18]
D	trimethoprim	5.60 μg/L	effluents	pharmaceutical industries area	Croatia	Bielen et al. [25]
D	trimethoprim	6.3 ng/L 22.1 ng/L 1.3 ng/L	Huangpu River Pearl River Estuary East China Sea	urban area from Dongjiang towards the open sea influenced by poultry and fish farming	China	Fisch et al. [34]
D	trimethorpim	380 ng/L	river water	Pearl River System	China	Yang et al. [35]
F	ciprofloxacin	32.8 µg/kg 1290 µg/kg 28.7 µg/kg	sediments sediments sediments	Yellow River Hai River Liao River	China	Zhou et al. [16]
F	ciprofloxacin	176.14 ng/L	drinking water	Yangtze River delta	China	Cui et al. [17]
F	ciprofloxacin	641.3 ng/L	river water	Beijing-Tianjin-Hebei region	China	Cheng et al. [18]
F	ciprofloxacin	1.14 μg/L ND	wastewaters river water	different locations	Argentina	Teglia et al. [21]
F	ciprofloxacin	0.25 μg/L 592 µg/kg	pond effluents sediments	fish farm in the Mekong Delta	Vietnam	Andrieu et al. [28]
F	difloxacin	14.2 μg/L ND	wastewaters river water	different locations	Argentina	Teglia et al. [21]
F	enoxacin	22.1 μg/L ND	wastewaters river water	different locations	Argentina	Teglia et al. [21]
F	enrofloxacin	ND 2.34 µg/kg ND	sediments sediments sediments	Yellow River Hai River Liao River	China	Zhou et al. [16]
F	enrofloxacin	19.33 ng/L	drinking water	Yangtze River delta	China	Cui et al. [17]
F	enrofloxacin	5681.9 ng/L	river water	Beijing-Tianjin-Hebei region	China	Cheng et al. [18]
F	enrofloxacin	11.9 μg/L 0.97 μg/L	wastewaters river water	different locations	Argentina	Teglia et al. [21]
F	enrofloxacin	98.00 μg/L	effluents	pharmaceutical industries area	Croatia	Bielen et al. [25]
F	enrofloxacin	0.86 μg/L 2590 µg/kg	pond effluents sediments	fish farm in the Mekong Delta	Vietnam	Andrieu et al. [28]
F	enrofloxacin	1.64 ng/L	water samples	urban area of Beijing	China	Ma et al. [36]
F	fluoroquinolones	<0.02 µg/L 6 µg/kg	fresh water sediments	14 sampling points in Polish rivers and lakes	Poland	Gbylik-Sikorska et al. [22]
F	lomefloxacin	ND 298 µg/kg 5.82 µg/kg	sediments sediments sediments	Yellow River Hai River Liao River	China	Zhou et al. [16]
F	norfloxacin	141 µg/kg 5770 µg/kg 176 µg/kg	sediments sediments sediments	Yellow River Hai River Liao River	China	Zhou et al. [16]
F	norfloxacin	48.39 ng/L	drinking water	Yangtze River delta	China	Cui et al. [17]
F	norfloxacin	1893.2 ng/L	river water	Beijing-Tianjin- Hebei region	China	Cheng et al. [18]
F	norfloxacin	1.43 ng/L	water samples	urban area of Beijing	China	Ma et al. [36]
F	norfloxacin	174 ng/L	river water	Pearl River System	China	Yang et al. [35]
F	ofloxacin	123 µg/kg 653 µg/kg 50.5 µg/kg	sediments sediments sediments	Yellow River Hai River Liao River	China	Zhou et al. [16]
F	ofloxacin	18.95 ng/L	drinking water	Yangtze River delta	China	Cui et al. [17]
F	ofloxacin	11.2 ng/L	river water	Beijing-Tianjin-Hebei region	China	Cheng et al. [18]
F	ofloxacin	1.78 μg/L ND	wastewaters river water	different locations	Argentina	Teglia et al. [21]
F	ofloxacin	4.3 µg/L	river water	main rivers of Hong Kong	China	Deng et al. [37]
F	ofloxacin	2.34 ng/L	water samples	urban area of Beijing	China	Ma et al. [36]
L	lincosamides	<0.02 µg/L ND	fresh water sediments	14 sampling points in Polish rivers and lakes	Poland	Gbylik-Sikorska et al. [22]
M	anhydro-erythromycin	49 ng/L	groundwater	monitoring wells in Baden-Württemberg	Germany	Sacher et al. [23]
M	azithromycin	≈450 ng/L ND	Tamagawa River (water samples) Mekong Delta (water samples)	Tokyo metropolitan area suburbs and rural areas	Japan Vietnam	Managaki et al. [24]
M	azithromycin	3776.00 μg/L	effluents	pharmaceutical industries area	Croatia	Bielen et al. [25]
M	erythromycin-H_2_O	49.8 µg/kg 67.7 µg/kg 40.3 µg/kg	sediments sediments sediments	Yellow River Hai River Liao River	China	Zhou et al. [16]
M	erythromycin-H_2_O	≈120 ng/L ≈50 ng/L	Tamagawa River (water samples) Mekong Delta (water samples)	Tokyo metropolitan area suburbs and rural areas	Japan Vietnam	Managaki et al. [24]
M	erythromycin-H_2_O	2009.00 μg/L	effluents	pharmaceutical industries area	Croatia	Bielen et al. [25]
M	erythromycin-H_2_O	2070 ng/L	river water	Pearl River System	China	Yang et al. [35]
M	macrolides	5 µg/L 8 µg/kg	fresh water sediments	14 sampling points in Polish rivers and lakes	Poland	Gbylik-Sikorska et al. [22]
M	*N*-Desmethyl azithromycin	5660.00 μg/L	effluents	pharmaceutical industries area	Croatia	Bielen et al. [25]
M	roxithromycin	6.8 µg/kg 11.7 µg/kg 29.6 µg/kg	sediments sediments sediments	Yellow River Hai River Liao River	China	Zhou et al. [16]
M	roxithromycin	≈50 ng/L ND	Tamagawa River (water samples) Mekong Delta (water samples)	Tokyo metropolitan area suburbs and rural areas	Japan Vietnam	Managaki et al. [24]
M	roxithromycin	2.35 ng/L	drinking water	Yangtze River delta	China	Cui et al. [17]
M	roxithromycin	1880 ng/L	river water	Pearl River System	China	Yang et al. [35]
M	clarithromycin	≈250 ng/L ND	Tamagawa River (water samples) Mekong Delta (water samples)	Tokyo metropolitan area suburbs and rural areas	Japan Vietnam	Managaki et al. [24]
P	pleuromutilins	<0.02 µg/L ND	fresh water sediments	14 sampling points in Polish rivers and lakes	Poland	Gbylik-Sikorska et al. [22]
Ph	chloramphenicol	5.8 ng/L	river water	Beijing-Tianjin-Hebei region	China	Cheng et al. [18]
Ph	florfenicol	1784.7 ng/L	river water	Beijing-Tianjin-Hebei region	China	Cheng et al. [18]
Ph	thiamphenicol	13.1 ng/L	river water	Beijing-Tianjin-Hebei region	China	Cheng et al. [18]
S	sulfachloropyridazine	3.49 ng/L	drinking water	Yangtze River delta	China	Cui et al. [17]
S	sulfadiazine	0.29 µg/L 1.00 µg/L 0.89 µg/L 17.0 µg/L	pond water river water farm effluents animal wastewater	27 large-scale animal farms (Jiangsu Province)	China	Wei et al. [15]
S	sulfadiazine	22.0 µg/kg 1.18 µg/kg 11 µg/kg	sediments sediments sediments	Yellow River Hai River Liao River	China	Zhou et al. [16]
S	sulfadiazine	20.82 ng/L	drinking water	Yangtze River delta	China	Cui et al. [17]
S	sulfadiazine	4.7 ng/L	river water	Beijing-Tianjin-Hebei region	China	Cheng et al. [18]
S	sulfadiazine	20.00 μg/L	effluents	pharmaceutical industries area	Croatia	Bielen et al. [25]
S	sulfadiazine	14.8 µg/L	river water	main rivers of Hong Kong	China	Deng et al. [37]
S	sulfadiazine	14.5 ng/L 3.0 ng/L 8.3 ng/L	Huangpu River Pearl River Estuary East China Sea	urban area from Dongjiang towards the open sea influenced by poultry and fish farming	China	Fisch et al. [34]
S	sulfadiazine	3.17 ng/L	water samples	urban area of Beijing	China	Ma et al. [36]
S	sulfadiazine	103 ng/L	river water	Pearl River System	China	Yang et al. [35]
S	sulfadimidine	580.4 µg/L	river water	main rivers of Hong Kong	China	Deng et al. [37]
S	sulfadoxine	0.29 µg/L 0.46 µg/L 0.10 µg/L 0.63 µg/L	pond water river water farm effluents animal wastewater	27 large-scale animal farms (Jiangsu Province)	China	Wei et al. [15]
S	sulfamerazine	ND 11.9 ng/L ND	Huangpu River Pearl River Estuary East China Sea	urban area from Dongjiang towards the open sea influenced by poultry and fish farming	China	Fisch et al. [34]
S	sulfamethazine	0.64 µg/L 4.66 µg/L 169 µg/L 211 µg/L	pond water river water farm effluents animal wastewater	27 large-scale animal farms (Jiangsu Province)	China	Wei et al. [15]
S	sulfamethazine	ND 5.67 µg/kg ND	sediments sediments sediments	Yellow River Hai River Liao River	China	Zhou et al. [16]
S	sulfamethazine	ND ≈350 ng/L	Tamagawa River (water samples) Mekong Delta (water samples)	Tokyo metropolitan area suburbs and rural areas	Japan Vietnam	Managaki et al. [24]
S	sulfamethazine	9.60 µg/L 0.28 µg/kg	water sediments	Naerincheon River across Hongcheon, Gangwon province	Korea	Awad et al. [5]
S	sulfamethazine	14.50 ng/L	drinking water	Yangtze River delta	China	Cui et al. [17]
S	sulfamethazine	3.8 ng/L	river water	Beijing-Tianjin-Hebei region	China	Cheng et al. [18]
S	sulfamethazine	231.00 μg/L	effluents	pharmaceutical industries area	Croatia	Bielen et al. [25]
S	sulfamethazine	446 ng/L	river water	Pearl River System	China	Yang et al. [35]
S	sulfamethoxazole	0.19 µg/L 0.56 µg/L 0.57 µg/L 63.6 µg/L	pond water river water farm effluents animal wastewater	27 large-scale animal farms (Jiangsu Province)	China	Wei et al. [15]
S	sulfamethoxazole	410 ng/L	groundwater	monitoring wells in Baden-Württemberg	Germany	Sacher et al. [23]
S	sulfamethoxazole	≈40 ng/L ≈180 ng/L	Tamagawa River (water samples) Mekong Delta (water samples)	Tokyo metropolitan area suburbs and rural areas	Japan Vietnam	Managaki et al. [24]
S	sulfamethoxazole	0.44 µg/L 0.73 µg/kg	water sediments	Naerincheon River across Hongcheon, Gangwon province	Korea	Awad et al. [5]
S	sulfamethoxazole	51.86 ng/L	drinking water	Yangtze River delta	China	Cui et al. [17]
S	sulfamethoxazole	11.6 ng/L	river water	Beijing-Tianjin-Hebei region	China	Cheng et al. [18]
S	sulfamethoxazole	3.1 µg/L	river water	main rivers of Hong Kong	China	Deng et al. [37]
S	sulfamethoxazole	9.6 ng/L 13.9 ng/L 4.4 ng/L	Huangpu River Pearl River Estuary East China Sea	urban area from Dongjiang towards the open sea influenced by poultry and fish farming	China	Fisch et al. [34]
S	sulfamethoxazole	1.82 ng/L	water samples	urban area of Beijing	China	Ma et al. [36]
S	sulfamethoxazole	418 ng/L	river water	Pearl River System	China	Yang et al. [35]
S	sulfapyridine	≈140 ng/L ND	Tamagawa River (water samples) Mekong Delta (water samples)	Tokyo metropolitan area suburbs and rural areas	Japan Vietnam	Managaki et al. [24]
S	sulfapyridine	3.2 µg/L	river water	main rivers of Hong Kong	China	Deng et al. [37]
S	sulfapyrindine	41.7 ng/L	river water	Pearl River System	China	Yang et al. [35]
S	sulfaquinoxaline	ND det det 0.64 µg/L	pond water river water farm effluents animal wastewater	27 large-scale animal farms (Jiangsu Province)	China	Wei et al. [15]
S	sulfathiazole	10.57 µg/L 0.64 µg/kg	water sediments	Naerincheon River across Hongcheon, Gangwon province	Korea	Awad et al. [5]
S	sulfonamides	<0.05 µg/L 5 µg/kg 10 µg/kg	fresh water sediments fish	14 sampling points in Polish rivers and lakes	Poland	Gbylik-Sikorska et al. [22]
S	sulfamerazine	2.49 ng/L	water samples	urban area of Beijing	China	Ma et al. [36]
T	chlortetracycline	49.3 ng/L	river water	Beijing-Tianjin-Hebei region	China	Cheng et al. [18]
T	chlortetracycline	0.57 µg/L 2.42 µg/L 3.67 µg/L 1.10 µg/L	pond water river water farm effluents animal wastewater	27 large-scale animal farms (Jiangsu Province)	China	Wei et al. [15]
T	chlortetracycline	ND 10.9 µg/kg 32.5 µg/kg	sediments sediments sediments	Yellow River Hai River Liao River	China	Zhou et al. [16]
T	chlortetracycline	44.42 µg/L 16.30 µg/kg	water sediments	Naerincheon River across Hongcheon, Gangwon province	Korea	Awad et al. [5]
T	oxytetracycline	6.87 µg/L 2.20 µg/L 11.1 µg/L 72.9 µg/L	pond water river water farm effluents animal wastewater	27 large-scale animal farms (Jiangsu Province)	China	Wei et al. [15]
T	oxytetracycline	184 µg/kg 422 µg/kg 652 µg/kg	sediments sediments sediments	Yellow River Hai River Liao River	China	Zhou et al. [16]
T	oxytetracycline	0.32 µg/L 1.43 µg/kg	water sediments	Naerincheon River across Hongcheon, Gangwon province	Korea	Awad et al. [5]
T	oxytetracycline	241.50 ng/L	drinking water	Yangtze River delta	China	Cui et al. [17]
T	oxytetracycline	51.5 ng/L	river water	Beijing-Tianjin-Hebei region	China	Cheng et al. [18]
T	oxytetracycline	29.00 μg/L	effluents	pharmaceutical industries area	Croatia	Bielen et al. [25]
T	tetracycline	0.93 µg/L 0.81 µg/L 6.44 µg/L 10.3 µg/L	pond water river water farm effluents animal wastewater	27 large-scale animal farms (Jiangsu Province)	China	Wei et al. [15]
T	tetracycline	18.0 µg/kg 135 µg/kg 4.82 µg/kg	sediments sediments sediments	Yellow River Hai River Liao River	China	Zhou et al. [16]
T	tetracycline	254.82 µg/L 75.70 µg/kg	water sediments	Naerincheon River across Hongcheon, Gangwon province	Korea	Awad et al. [5]
T	tetracycline	94.66 ng/L	drinking water	Yangtze River delta	China	Cui et al. [17]
T	tetracycline	31.4 ng/L	river water	Beijing-Tianjin-Hebei region	China	Cheng et al. [18]
T	tetracycline	26 ng/L	water samples	urban area of Beijing	China	Ma et al. [36]
T	tetracyclines	0.05 µg/L 5 µg/kg	fresh water sediments	14 sampling points in Polish rivers and lakes	Poland	Gbylik-Sikorska et al. [22]
T	doxycycline	ND ND ND 39.5 µg/L	pond water river water farm effluents animal wastewater	27 large-scale animal farms (Jiangsu Province)	China	Wei et al. [15]
T	doxycycline	ND 7.0 µg/kg 2.8 µg/kg	sediments sediments sediments	Yellow RiverHai RiverLiao River	China	Zhou et al. [16]
T	doxycycline	43.3 ng/L	river water	Beijing-Tianjin-Hebei region	China	Cheng et al. [18]
T	doxycycline	82.2 µg/L	river water	main rivers of Hong Kong	China	Deng et al. [37]

det—detected, quantification not possible; ND—not detected, A—aminoglycosides, BL—beta lactams, D—diaminopyrimidines, F—fluoroquinolones, L—lincosamides, M—macrolides, P—pleuromutulins, Ph—phenicol antibiotics, S—sulfonamides, T—tetracyclines.

## 3. Antibiotic Resistance in Aquatic Environment

Increasing antibiotic resistance of bacterial fish pathogens in aquaculture is observed for many years [57,58,59]. Natural ecosystems, including aquatic environment, constitute a potential source of antibiotic resistance genes (ARGs) for human pathogens [60]. Recently, it was recognized that antibiotic resistant microorganisms and resistance determinants are ubiquitous in environments, even those that were never exposed to antimicrobial agents, and that environment is an important reservoir of emerging antibiotic resistance genes [29]. The ARGs evolved over time under natural conditions, long before the use of antibiotics [61]. However, irrational use of antibiotics in the last four decades accelerated spread of antibiotic resistant bacteria across the globe [59]. Antibiotics are used in aquaculture to prevent and treat bacterial infections in fish and invertebrates [58]. Excessive use of antibiotics in aquaculture results from frequent bacterial infections in fish under conditions of intensive culture. Antibiotics present for a long time at significant concentrations in aquatic environment are the principal selective pressure for antimicrobial resistance in bacteria. Susceptible bacteria in the sediment and water are replaced by resistant ones [58]. Antibiotics in fish culture are usually administered via feed and the intestinal environment of fish is an optimal site for the selection of antibiotic resistant bacteria [62]. Through fish feces, antibiotic resistant bacteria are dispersed to the water column or sediments. While, subinhibitory concentrations of antibiotics may stimulate mutagenesis and horizontal transfer of genes [63]. Mutations can lead to change of specific molecular structures in bacterial cells that are attacked by the antibiotics and loss of antibiotic affinity of these structures [59]. Resistance to antimicrobials may also result from transferring of resistance genes among the bacteria. Horizontal transfer of ARGs from one bacterium to another is a common mechanism [64]. Resistance of bacterial pathogens to antimicrobial treatment is not limited to the intensive culture of fish but also is a great challenge in human and veterinary medicine [65]. Aquaculture can promote the occurrence of antibiotic resistance in human and animal bacterial pathogens because aquaculture facilities are often recipients of animal manure and urine that may contain antibiotics and bacterial cells carrying AGRs [66]. The presence of antibiotics in manure results from their use as growth promoters for farm animals. Hospital wastewaters released into the aquatic environment are another major source of AGRs and antibiotic-resistant organisms [7]. ARGs could be acquired by pathogens via horizontal gene transfer in aquaculture environment and in the food chain leading to decreased efficacy of different antibiotic groups and severely limits the therapeutic options in human infections [67]. Another site of AGRs transfer are sewage treatment plants, where resistant bacteria and other pathogens present in wastewater remain in close contact with sewage sludge bacteria during biological treatment [68]. Horizontal gene transfer may also take place in biofilms that are present both in live aquatic organisms and on other surfaces [69]. Some of antimicrobial resistance genes were first detected in aquatic bacteria and later also among human and animal pathogens. These include some of the quinolone resistance genes occurring in *Vibrio*, *Shewanella,* and *Aeromonas*, β-lactamase genes which are presence in *Photobacterium damselae* and *Oceanobacillus iheyensis*, a novel fosfomycin resistance gene isolated from aquatic environment, and the chloramphenicol resistance genes from aquatic *Photobacterium*, *Vibrio,* and *Shewanella* [58]. Resistance gene variants for β-lactams, aminoglycosides, tetracyclines, macrolides were also detected in the genome of *Renibacteriun salmoninarum* and *Stenotrophomonas maltophilia* [70,71]. In aquaculture farms, where antibiotics were used, significant increase in the frequency of bacteria resistant to oxytetracycline, quinolones, sulfa/trimethoprim, florfenicol, and amoxicillin was observed [58]. Oxytetracycline is a broad-spectrum antibiotic, belonging to the tetracycline class and is one of the antibiotics most widely used in aquaculture. This antibiotic is used primarily in Canada, Norway, and the United States for treatment of fish bacterial diseases caused by *Aeromonas salmonicida*, *A. hydrophila*, *A. sobia*, *Pseudomonas*, *Lactococcus garvieae,* and *Vibrio anguillarum* [72]. Seyfried et al. [73] showed higher prevalence of tetracycline resistance (*tetR*) genes in aquaculture facilities with oxytetracycline use than those in which this antibiotic was not applied. Tamminen et al. [74] also showed the presence of tetracycline resistance genes such as *tetA, tetC, tetH,* and *tetM* in fish farm environments. Canada and Norway permit use of florfenicol, and Norway permits aquacultural use of quinolones. Florfenicol and oxytetracycline were the most frequently used antibiotics during 2016 in the Chilean salmon farms, which were mainly used to treat *Piscirickettsia salmonis*, currently considered as the main bacterial pathogen of salmon [29]. Fish pathogens and other aquatic bacteria such as *Aeromonas salmonicida*, *A. hydrophila*, *Citrobacter freundii*, *Vibrio salmonicida*, *Flavobacterium psychrophilum,* and *Pseudomonas fluorescens* developed resistance as a consequence of exposure to antibacterial agents [75]. The multidrug resistance of *A. salmonicida* pathogenic to fish is a result of the presence of transferable resistance plasmids in bacterial cells, and was described in many countries [76]. Akinbowale et al. [77] showed that among Gram-negative and Gram-positive bacteria isolated from farmed fish, crustaceans, and water from different parts of Australia, the resistance to β-lactams (ampicillin, amoxycillin), first-generation cephalosporins (cephalexin), and macrolides (erythromycin) was widespread. Additionally, resistance to oxytetracycline, tetracycline, nalidixic acid, and sulfonamides was common and multiple resistance was also observed. The study by Akinbowale et al. [77] provides support to the view that there is the risk of transfer of resistant bacteria to humans from consumption of aquaculture products. *Aeromonas* isolates from fish farms in Denmark showed resistance to amoxicillin (100%), oxytetracycline (69%), sulfadiazine-trimethoprim (43%), and oxolinic acid (20%). While, all *Flavobacterium psychrophilum* isolates were resistant to oxolinic acid, 71% and 50% were oxytetracycline and amoxicillin resistant, respectively [78]. *Aeromonas* isolates from three fish farms producing tilapia in South Africa were in 44% resistant to nalidixic acid and quinolone resistance genes: *qnrB* and *qnrS* were detected in 41% and 24% of isolates, respectively. The results obtained by Xiong et al. [79] showed that fish ponds in Guangdong, China were reservoirs of ARGs, and that the concentrations of tetracyclines, sulfonamides, and fluoroquinolones were as high as 446 μg/kg and 98.6 ng/L in sediment and water samples, respectively. Among resistance genes, the presence of tetracycline resistance genes (*tetM*, *tetO*, *tetW*, *tetS*, *tetQ*, *tetX*, *tetB/P*), sulfonamide resistance genes (*sul1*, *sul2*, *sul3*), and plasmid-mediated quinolone resistance genes (*oqxA*, *oqxB*, *aac (6′)-Ib*, and *qnrS*) was confirmed. Tamminen et al. [74] found that ARGs persisted at fish farms in the absence of antibiotic selection, which is a result of gene transfer through mobile genetic elements that remain for a long time in aquatic environment. Antibiotics entering aquatic environment favor the selection of antibiotic resistance among environmental bacteria and fish pathogens. From aquatic environment, antibiotic-resistant bacteria can be transferred to humans. Furthermore, antibiotics can accelerate horizontal AGRs transfer, increasing the probability of resistance gene transfer from environmental bacteria to human pathogenic bacteria.

## 4. Toxic Effects of Antibacterials on Fish Organisms

### 4.1. Hematological and Blood Biochemical Changes

Analysis of blood parameters is an important tool used to assess the toxic effects of xenobiotics on fish [80,81]. However, most available data concerning hematological effects of antibacterials in fish regard mainly oxytetracycline [82,83,84,85,86,87] and very little information about other antibacterials were found (Table 2). In all these studies fish were subjected to relatively high doses administered via diet or injection. Exposure of fish to antibiotics may induce various hematological changes—increase in red blood cell parameters (RBC, Ht, Hb) or anemic response (Table 2). Similarly, leukocyte count may decrease (usually) or increase (rarely). However, the data are scarce and thus it is impossible to formulate conclusions. 

Little is known also about the influence of antibacterials on the biochemical indices of plasma or serum, and the data published so far are mostly focused on the effects of oxytetracycline [82,83,84,88,89]. Analysis of the available data (Table 2) shows that activities of hepatic enzymes (ALT and AST) in fish exposed to oxytetracycline often increased, which may indicate a hepatotoxic effect of this antibiotic in fish. Hyperglycemia and an increase in cortisol show that antibiotics may induce stress response in fish.

**Table 2:** Effects of antibacterials on hematological and blood biochemical parameters in fish.

Antibacterial Agents	Dose	Exposure Time (days)	Species	Hematological or Blood Biochemical Alterations		Reference
				Increase	Decrease	
florfenicol	5 mg/kg body weight	84	*Oreochromis niloticus*	Lysoz, Urea	AST, Creat	Reda et al. [89]
gentamicin	36 mg/kg (injection)		*Oreochromis niloticus*	ALT, AST, Glu	Bicarb, Ca, Chol, CK Cl, Fe, Mg, Na, RBC, TIBC, TP	Chen et al. [90]
oxytetracycline	75 mg/kg body weight per day (dietary)	29	*Cyprinus carpio*	Ht	Glu, Lym, Mono, WBC	Dobsikova et al. [82]
oxytetracycline	75 mg/kg body weight per day (dietary)	50	*Cyprinus carpio*	Alb, Cl, Na, P, TP		Dobsikova et al. [82]
oxytetracycline	500 mg/kg of diet	14	*Oreochromis niloticus*	ALT, AST, Creat, PLT, Urea		El-Adawy et al. [83]
oxytetracycline	2.5 g/kg of diet	14	*Oncorhynchus mykiss*	ALT, AST, Cort, Glu	Lysoz, Neu, WBC	Hoseini and Yousefi [84]
oxytetracycline	100 mg/kg body weight per day (dietary)	14	*Oncorhynchus kisutch*	ALT, GSH		Nakano et al. [88]
oxytetracycline	0.63% of wet weight (dietary)	56	*Oreochromis niloticus*	Hb	Hb, PLT, RBC, WBC	Omoregie and Oyebanji [85]
oxytetracycline	1.25–5.00% of wet weight (dietary)	56	*Oreochromis niloticus*		Hb, Ht, PLT, RBC, WBC	Omoregie and Oyebanji [85]
oxytetracycline	100 mg/kg diet	84	*Oreochromis niloticus*	AST, Lysoz, Urea	ALT, Creat, IgM	Reda et al. [89]
oxytetracycline	0.5 g/kg	60	*Oreochromis niloticus*	PLT	Ht	Reda et al. [86]
oxytetracycline	75 mg/kg body weight	10	*Sparus aurata*	WBC		Serezli et al. [87]

Biochemical indices: Alb—albumins, ALT—alanine transaminase, AST—aspartate transaminase, Bicarb—bicarbonate, Ca—calcium, Chol—cholesterol, CK—creatine kinase, Cl—chlorine, Cort—cortisol, Creat—creatinine, Fe—iron, Glu—glucose, GSH—glutathione (in plasma), IgM—immunoglobulin M, Lysoz—lysozyme, Mg—magnesium, Na—sodium, P—phosphorus, TIBC—total iron blood capacity, TP—total protein, Urea—urea. Hematological indices: Hb—hemoglobin concentration, Ht—hematocrit, Lym—lymphocytes, Mono—monocytes, Neu—neutrophils, PLT—thrombocyte count, RBC—erythrocyte count, WBC—leukocyte count.

### 4.2. Oxidative Stress Parameters

Available data indicate that antimicrobials may induce oxidative stress in fish. Ni et al. [91] revealed that *Danio rerio* exposed to 0.1–2.5 mg/L of maduramicin for 14 days showed upregulation (0.1 mg/L) or inhibition (2.5 mg/L) of hepatic SOD (superoxide dismutase), CAT (catalase), GPx (glutathione peroxidase), and GST (glutathione s-transferase) accompanied by lipid peroxidation (increased MDA level). According to Elia et al. [92], *Cyprinus carpio* fed oxytetracycline at therapeutic level (75 mg/kg) or at high level (150 or 300 mg/kg) for 10 days exhibited impaired antioxidant system: hepatic SOD activity decreased, while activities of GPx and GR increased. Limbu et al. [93] fed *Oreochromis niloticus* 80 mg/kg of oxytetracycline for 35 days and reported decreased hepatic SOD and GST activities, while serum MDA level and AST activity increased which indicates oxidative damage. In addition, intestinal and hepatic caspase 9 was upregulated which indicates increased apoptotic activity. Increased expression of hepatic mRNA for aminopeptidases, fatty acid synthase, lipase, and carnitine palmitoyltransferase were also observed. The observed changes indicate that oxytetracycline induced oxidative stress and disturbed hepatic functions. Contrary, no such effects were observed for gentamicin treatment. Bojarski et al. [94] revealed that single injection of 5 mg/kg of gentamicin administered to *Carassius gibelio* did not induce alterations in GSH level or activities of SOD, GPx or CAT which indicates that no oxidative stress occurred. However, according to Jones et al. [95], various fish species may differ in sensitivity to gentamicin and other antibacterials due to different renal structure (glomerular vs. aglomerular nephrons) and thus different rates of excretion. According to Silva de Oliveira et al. [96] embryos of *Danio rerio* exposed for 168 h to 20 µg/L of nitrofurantoin showed biochemical changes: increase in ChE (cholinesterase), LDH (lactate dehydrogenase) and GST activities, while at 320 µg/L increase in CAT activity. Similarly, Nogueira et al. [97] reported that 96 h exposure to ciprofloxacin (0.005–0.488 µg/L) induced an increase in AChE activity and decrease in CAT activity in *Danio rerio* embryos or larvae. These data indicate that exposure of fish to antibacterials leads to changes in the oxidoreductive balance, and the type and magnitude of changes probably depend primarily on the type of therapeutic substance used and its dose, as well as fish species and developmental stage.

### 4.3. Histopathological Alterations

Histological analysis is often used to assess the toxic effects of xenobiotics on fish [94,98]. Rodrigues et al. [99] demonstrated that *Oncorhynchus mykiss* subjected to acute (96 h at 0.005–50 mg/L) or chronic (28 days at 0.31–5 µg/L) exposure to oxytetracycline showed minor or moderate and reversible histopathological lesions. Hypertrophy of mucous cells and hyperplasia of epithelial cells were observed in gills after acute exposure and lamellar fusion, epithelial lifting after chronic exposure. In liver, hemorrhage and increase of sinusoidal space, hepatocyte hypertrophy and nucleus pyknosis, vacuolation, and hepatocellular degeneration were observed after acute exposure. Islam et al. [100] revealed that *Barbonymus gonionotus* fed 4 g/kg of oxytetracycline for 45 days showed hepatic and renal histopathological lesions: vacuolation of hepatocytes, fatty degeneration of liver, and lymphocyte aggregations in kidney. 

The results obtained by Rodrigues et al. [101] demonstrated that *Oncorhynchus mykiss* exposed for 96 h to 0.001–10 mg/L or for 28 days to 0.05–0.8 µg/L of erythromycin showed various histopathological alterations: branchial epithelial hyperplasia and hypertrophy of mucous cells. After acute exposure aneurysm, edema, epithelial lifting, and lamellar fusion were also reported, accompanied by hepatocyte vacuolation, nuclear pyknosis, hepatocyte degeneration, sinusoid enlargement, and hemorrhage.

According to Augusto et al. [102], *Oreochromis nilotica* treated with gentamicin (2 intraperitoneal injections of 5 or 25 mg/kg) showed dose-dependent acute tubular necrosis. Regeneration of damaged epithelia and development of new nephrons were observed post injury. Similarly, Reimschuessel et al. [103] reported that *Opsanus tau* intraperitoneally injected with 2.5–50 mg/kg of gentamicin showed extensive necrosis of nephric proximal tubules and no recovery was observed. According to Reimschuessel and Williams [104], *Carassius auratus* after single intraperitoneal gentamicin dose of 50 mg/kg showed nephron injury followed by regeneration and development of new nephrons within 2–3 weeks post injection. Salice et al. [105] found nephron neogenesis in *Carassius auratus* subjected to repeated injections with 50 mg/kg of gentamicin. The above data clearly indicate that gentamicin is nephrotoxic to fish. 

These data show that fish exposure to various antibiotics can result in the development of histopathological lesions. The organs that appear to be most sensitive to the toxic effects of these substances are liver, kidneys, and gills.

### 4.4. Other Toxic Effects

Lethal concentrations of antibiotics to adult fish are usually high but different agents differ in their toxic power. According to Marking et al. [106], the values of 96 h LC_50_ of oxytetracycline, tetracycline and erythromycin for *Salvelinus namaycush* were: <200, 220, and 410 mg/L, respectively. Toxicity of 7 fluoroquinolone antibiotics to *Pimephales promelas* was studied and the results showed that only clinafloxacin caused almost 100% mortality at the concentration of 10 mg/L but no mortality at 2 mg/L [107]. Carraschi et al. [108] reported that 48 h LC_50_ of oxytetracycline to *Piaractus mesopotamicus* was 7.6 mg/L, while of florfenicol >1000 mg/L. According to Martins et al. [109], 96 h LC_50_ of ciprofloxacin to *Gambusia holbrooki* was >60 mg/L.

However, various sublethal effects were observed at low, environmentally realistic concentrations of antibiotics. Almeida et al. [110] reported enhanced exploratory behavior and stress response in *Danio rerio* after long-term exposure to low oxytetracycline concentrations. The authors supposed that antibiotic increased energy consumption and reduced lipid level. Oxidative damage was also observed and changes in the intestinal microbiota community structure. Toxic effects were observed at 0.1 µg/L which is an environmentally realistic concentration. According to Botelho et al. [111], *Oreochromis niloticus* exposed for 96 h to waterborne florfenicol and oxytetracycline at concentrations 212.5–850 or 4000–16000 ng/L, respectively (from the half to double environmental concentrations) showed genotoxic damage detected using comet assay and inspection of nuclear anomalies in erythrocytes. Oxytetracycline at low environmentally realistic concentrations induced glutathione pathway of detoxification that interfered with neurotransmission and caused metabolic disturbances in *Oncorhynchus mykiss* [112]. According to Zhou et al. [113], exposure of *Danio rerio* for 6 weeks to 0.26 µg/L of sulfamethoxazole or 0.42 µg/L of oxytetracycline caused an increase in fish metabolic rate and increased mortality due to *Aeromonas hydrophila* experimental infection. Exposure to antibiotics resulted in a decrease in intestinal goblet cell abundance, activities of alkaline and acid phosphatase, reduced antioxidant and increased inflammatory response, and oxytetracycline disturbed alimentary tract microbiota. Wang et al. [114] observed that β-diketone antibiotics at 6.25 or 12.5 mg/L reduced the levels of estradiol and testosterone, as well as gonadosomatic index of *Danio rerio* in a dose-dependent way. Egg production, fertilization rate, and hatching rate also declined. These results indicate reproductive toxicity of β-diketone antibiotics in fish.

Adverse effects of antibiotics on fish immune response were reported at both environmental concentrations and therapeutic doses of antibacterial compounds. According to Grondel et al. [115], isolated B and T pronephric lymphocytes of *Cyprinus carpio* after 4 days of incubation at 0.62–20 mg/L oxytetracycline and doxycycline showed disturbed mitotic activity, while no significant effect was observed at 0.15–0.31 mg/L. Rijkers et al. [116] observed suppression of humoral immune response in *Cyprinus carpio* treated orally with 2000 mg/kg of oxytetracycline, while in fish injected intraperitoneally (60 or 180 mg/kg) both humoral and cellular immune response were suppressed. Intramuscular injections of oxytetracycline and benzylpenicillin (20 mg/kg) disturbed homeostasis in healthy *Oncorhynchus mykiss* and caused immunosuppression [117].

Early developmental stages of fish are particularly sensitive to toxicity. Effluents from two Croatian pharmaceutical industries containing azithromycin or mixture (fluoroquinolones, trimethoprim, sulfonamides, and tetracyclines) caused embryotoxicity in *Danio rerio* manifested as increased mortality, delayed development, reduced hatching rate, body malformations such as cardiac and vitelline edema, scoliosis, lack of pigmentation, and functional anomalies-altered heartbeat rate [25]. Exposure for 4 days to 0.2–0.6 mg/L of triclosan decreased body length, head, and eye size in *Danio rerio* embryos and activated apoptosis in central nervous system which resulted in decreased synaptic density and decrease in axon length [118]. Yan et al. [119] reported that exposure of *Danio rerio* embryos to macrolide antibiotics (azithromycin, clarithromycin, tilmicosin, and tylosin) resulted in cardiotoxicity: increased heart rate and pericardial edema. It was accompanied by spine curvatures, increased apoptosis rate, and oxidative stress. Exposure for 24 h to fluoroquinolone antibiotics: gatifloxacin (413–4239 mg/L) and ciprofloxacin (156–1949 mg/L) reduced heart rate and cardiac output in *Danio rerio* embryos. Additionally, gatifloxacin exposure resulted in pericardial edema, while ciprofloxacin did not induce any morphological anomalies [120].

## 5. Conclusions

A wide variety of antimicrobial compounds are detected in aquatic environment, including antibiotics and chemotherapeutics. Although antimicrobials are used in aquaculture, they mostly come from terrestrial sources, mainly poultry and swine farm-effluents, and in lesser extent from human medicine. Concentrations of antimicrobial drugs in waters are usually very low (several ng or µg/L, very rarely reach the range of mg/L), being below lethal levels for fish. Most data concerning toxicity of antimicrobials to fish involve therapeutic doses that often produce side effects. However, some reports indicate that chronic exposure of fish to low, environmentally realistic concentrations of antimicrobial agents may result in physiological disturbances such as hematological alterations, oxidative stress, histopathological lesions, immunosuppression, metabolic disorders, genotoxic damage, general stress response, and reproductive impairment.

Disturbances in aquatic bacterial communities and symbiotic (particularly alimentary tract) microbiota of fish are another possible effect water contamination with antimicrobials. 

Emergence of antibiotic-resistant pathogenic bacteria in aquatic environment is also a serious threat due to selective pressure of subinhibitory concentrations of antibiotics present in water that accelerate transfer and spread of antibiotic resistance genes in bacterial communities. 
